# Heritability of Cardiovascular and Personality Traits in 6,148 Sardinians

**DOI:** 10.1371/journal.pgen.0020132

**Published:** 2006-08-25

**Authors:** Giuseppe Pilia, Wei-Min Chen, Angelo Scuteri, Marco Orrú, Giuseppe Albai, Mariano Dei, Sandra Lai, Gianluca Usala, Monica Lai, Paola Loi, Cinzia Mameli, Loredana Vacca, Manila Deiana, Nazario Olla, Marco Masala, Antonio Cao, Samer S Najjar, Antonio Terracciano, Timur Nedorezov, Alexei Sharov, Alan B Zonderman, Gonçalo R Abecasis, Paul Costa, Edward Lakatta, David Schlessinger

**Affiliations:** 1 Istituto di Neurogenetica e Neurofarmacologia, Consiglio Nazionale delle Ricerche, Ospedale Microcitemico, Cagliari, Italy; 2 Center for Statistical Genetics, Department of Biostatistics, University of Michigan, Ann Arbor, Michigan, United States of America; 3 Unità Operativa Geriatria, Istituto Nazionale Riposo e Cura Anziani, Rome, Italy; 4 Gerontology Research Center, National Institute on Aging, Baltimore, Maryland, United States of America; 5 Unità Operativa Semplice Cardiologia, Divisione di Medicina, Presidio Ospedaliero Santa Barbara, Iglesias, Italy; The Jackson Laboratory, United States of America

## Abstract

In family studies, phenotypic similarities between relatives yield information on the overall contribution of genes to trait variation. Large samples are important for these family studies, especially when comparing heritability between subgroups such as young and old, or males and females. We recruited a cohort of 6,148 participants, aged 14–102 y, from four clustered towns in Sardinia. The cohort includes 34,469 relative pairs. To extract genetic information, we implemented software for variance components heritability analysis, designed to handle large pedigrees, analyze multiple traits simultaneously, and model heterogeneity. Here, we report heritability analyses for 98 quantitative traits, focusing on facets of personality and cardiovascular function. We also summarize results of bivariate analyses for all pairs of traits and of heterogeneity analyses for each trait. We found a significant genetic component for every trait. On average, genetic effects explained 40% of the variance for 38 blood tests, 51% for five anthropometric measures, 25% for 20 measures of cardiovascular function, and 19% for 35 personality traits. Four traits showed significant evidence for an X-linked component. Bivariate analyses suggested overlapping genetic determinants for many traits, including multiple personality facets and several traits related to the metabolic syndrome; but we found no evidence for shared genetic determinants that might underlie the reported association of some personality traits and cardiovascular risk factors. Models allowing for heterogeneity suggested that, in this cohort, the genetic variance was typically larger in females and in younger individuals, but interesting exceptions were observed. For example, narrow heritability of blood pressure was approximately 26% in individuals more than 42 y old, but only approximately 8% in younger individuals. Despite the heterogeneity in effect sizes, the same loci appear to contribute to variance in young and old, and in males and females. In summary, we find significant evidence for heritability of many medically important traits, including cardiovascular function and personality. Evidence for heterogeneity by age and sex suggests that models allowing for these differences will be important in mapping quantitative traits.

## Synopsis

Genetic analysis of complex traits, which are influenced by many different variables, is difficult because different genes and environmental factors can affect each individual. To simplify analysis, the authors turned to Sardinia, one of the rare, relatively isolated populations. They recruited 6,148 participants, aged 14–102 y, from four neighboring towns. Their sample includes many related individuals, including, for example, approximately 5,000 pairs of brothers and sisters. The authors measured 98 traits in each individual, including different aspects of blood composition and several cardiovascular and personality measures.

Here, the authors evaluate the overall impact of genes and environment on each trait and show that genes can explain many of the differences and similarities between individuals. Genetic influences were typically larger in females and in younger individuals, but interesting exceptions were observed. For example, genetic factors accounted for approximately 26% of the variation in blood pressure for those more than 42 y old, but only for approximately 8% in younger individuals. Their analysis also showed that the same genetic factor could influence multiple traits, for example by affecting multiple features of personality or of cardiovascular function. DNA analyses of this cohort will eventually allow researchers to identify genes that affect each of the traits studied, including important risk factors for cardiovascular disease.

## Introduction

Complex traits, including aging-associated conditions, can be influenced by a multiplicity of genetic and environmental factors. Because each factor is expected to make only a small contribution to trait variability, and this contribution may itself be influenced by interactions with other susceptibility factors, identifying the genetic basis of complex traits is challenging and requires large sample sizes [1]. Isolated founder populations, which have already proven useful in the study of many Mendelian disorders [2], provide an attractive setting for the study of complex traits [3,4] because they typically exhibit greater genetic and environmental homogeneity than more cosmopolitan populations.

Sardinia is the second largest island in the Mediterranean. Its modern population numbers approximately 1.65 million and constitutes a genetically isolated founder population [5–7], which has already aided in the identification of genes involved in several Mendelian disorders [8–12]. In addition to its status as an isolated founder population and its relatively large size, the Sardinian population is attractive for genetic studies due to its organization into long-established settlements [13].

Here, we use a large cohort of 6,148 Sardinians to study the heritability of a spectrum of 98 quantitative traits. Studying broad groups of traits, we could assess the generality of any trends, such as changes in heritability with aging. To increase the potential clinical utility of the results, we focused on traits that affect major domains of clinical interest. For example, in addition to anthropometric features, we quantified levels of plasma and serum markers, including total cholesterol, high-density lipoprotein (HDL), and low-density lipoprotein (LDL) levels, and measured subclinical vascular alterations [14–18] that are of intrinsic interest and are also useful predictors of cardiovascular disease [19]. Similarly, we assessed individual differences in personality using the five-factor model [20,21], which quantifies recurring dimensions of personality. Again, in addition to their intrinsic interest, these personality traits are important in understanding a variety of important life outcomes, including mental disorders.

Our study uses the full range of phenotypic variation in the population to dissect the genetic contribution and provide a quantitative assessment of the impact of inherited variation on each trait. In addition, we report evidence for heterogeneity in the genetic and environmental contributions to variation, by comparing variances and covariances between males and females and between the younger and older individuals in our cohort. Finally, we examine evidence for an overlap in the genetic determinants of multiple traits, identifying clusters of traits that appear to be influenced by the same genes. The joint study of cardiovascular and personality traits afforded us an opportunity to look for a genetic factor that might contribute to the association of certain personality traits and cardiovascular problems [22]. Overall, our results should be useful to investigators interested in identifying the genetic determinants of quantitative trait variation, especially for clinically relevant quantitative traits affecting cardiovascular function and personality.

## Results

### Cohort Recruitment

**Figure 1:**
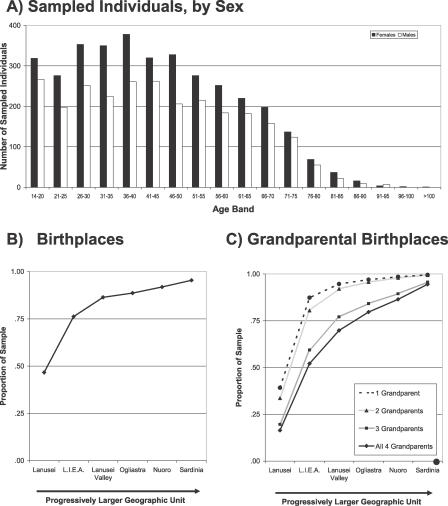
Age, Sex, and Birthplace Distribution for Participants (A) Shows the number of recruited females (black bars) and males (white bars) from the four clustered towns. (B) Shows the birthplace distribution of participants, in progressively larger geographic units: Lanusei, L.I.E.A. (Lanusei and the three surrounding towns of Ilbono, Elini, and Arzana), the Lanusei valley, the region of Ogliastra, the province of Nuoro, and all of Sardinia. (C) Shows the birthplace distribution for grandparents of participants in the same progressively larger geographic units.

We recruited and phenotyped 6,148 individuals, male and female, age 14 y and above (Figure 1A) from a cluster of four towns in the Lanusei Valley in the Ogliastra region of the Sardinian province of Nuoro. This corresponds to approximately 62% of the population eligible for recruitment in the area, which totaled 9,841 individuals in the 2001 census. Compared to the census population, our sample is enriched for females at all ages (3,523 individuals, or 57%, of our sample, compared to 5,089, or 52%, of the census population). Ascertainment was less complete for individuals more than 74 y of age, among whom only approximately 29% of the population was recruited (238 individuals more than 74 y recruited, but 813 were reported in the 2001 census).

Nearly all subjects were born in Sardinia (5,857 [95%]) and, specifically, in the Ogliastra region (5,442 [89%]; Figure 1B shows the birth places of participants in the restricted geographical region). Emphasizing the stability of the population, all grandparents were born in Sardinia for 95% of participants (Figure 1C). The cohort is organized into multiple complex pedigrees. Information collected at recruitment allowed us to organize 5,610 individuals into 711 connected pedigrees, each up to five generations deep. The largest pedigree connects 625 phenotyped individuals. In total the sample includes 34,469 relative pairs, with an average kinship coefficient of 0.1628. These relative pairs include 4,933 sibling pairs, 180 half-sibling pairs, 4,014 first cousins, 4,256 parent–child pairs, 675 grandparent–grandchild pairs, and 6,400 avuncular pairs in addition to other more distant relatives. Our sample also includes 11 monozygotic twins (identified by genotyping approximately 10,000 single nucleotide polymorphisms in all individuals). Because monozygotic twins are often more similar to each other than predicted by a simple genetic model (even with genetic dominance included), we included only one individual from each of these twin pairs in the analysis reported below.

### Summary of Quantitative Trait Variation

**Figure 2:**
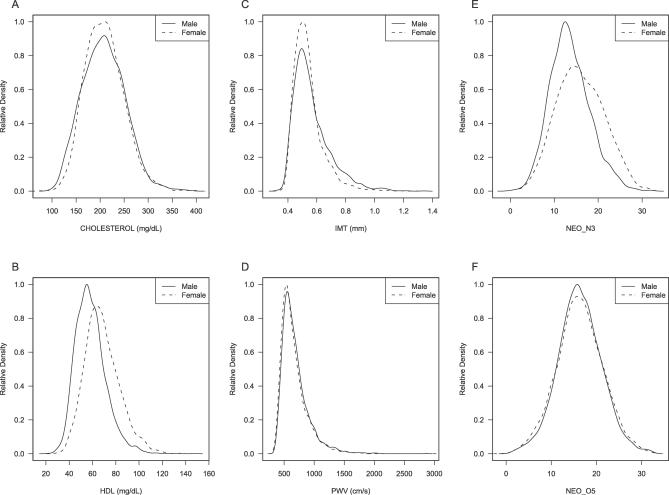
Distribution of Six Illustrative Traits in Male and Female Participants Relative densities are plotted for males (solid lines) and females (dashed lines) for two serum values (cholesterol levels [A] and HDL [B]), two measures of cardiovascular function (IMT of the carotid artery [C] and PWV [D]), and two personality facets (NEO_N3 [E] and NEO_O5 [F]). A complete set of plots, including all traits, is available online (http://www.sph.umich.edu/csg/chen/public/sardinia).

**Figure 3:**
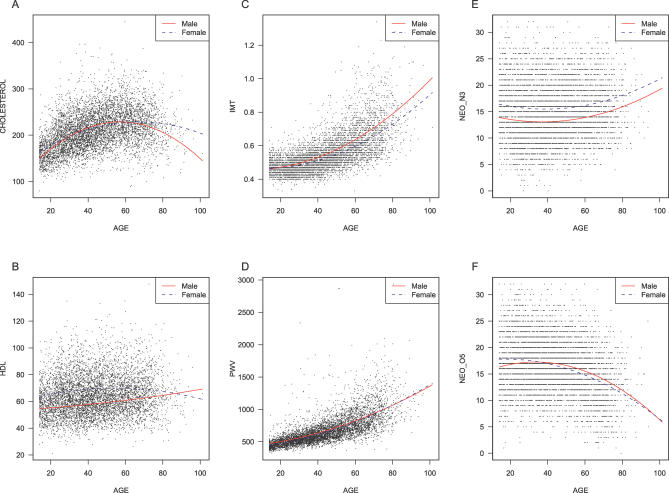
Illustrative Quantitative Traits Plotted as a Function of Age These are the same traits as in Figure 2. All values are plotted, and polynomial regression curves fitted to the data show inferred trends for males (solid red lines) and females (dashed blue lines) with increasing age. A complete set of plots, allowing for all traits, is available online (http://www.sph.umich.edu/csg/chen/public/sardinia).

To examine the effect of age and sex on each trait, we first generated and reviewed summary plots for each trait. The complete set of plots is available online (http://www.sph.umich.edu/csg/chen/public/sardinia) together with detailed results for all our analysis. Figure 2 displays the distribution of six illustrative traits for males and females. It is clear that for many traits there are marked differences between the sexes, affecting not only trait means, but also the overall pattern of variability around these means. Figure 3 illustrates the effect of age on the same six traits. For each trait, observed measurements are plotted against age at enrollment, and two quadratic regression lines (blue for females and red for males) are presented to summarize the impact of age on the traits. These plots allowed us to identify outliers in each trait and to compare trait distributions with other studies.

We next calculated the mean and standard deviation for all traits, both in the entire cohort and after stratifying the sample by sex and age. When stratifying the sample by age, we considered four age bands (14–29, 30–44, 45–59, and 60–102 y of age), each including approximately 25% of sampled individuals. The results are summarized in Table S1, with traits organized as blood test results (38 traits), anthropometric measures (five traits), cardiovascular measures (20 traits), and personality traits (five factors and 30 facets of personality). Nearly all traits showed highly significant evidence (analysis of variance *p* < 0.0005) for differences in trait means between the sexes (75 of 98 traits) and across age bands (91 of 98 traits).

### Estimates of Quantitative Trait Heritability

We next used quantile normalization to convert each trait to approximate normality, and fitted a simple model with two variance components (a heritable additive polygenic component and an individual specific environmental component) and five covariates (sex, age, age^2^, and the terms for the interaction of sex with age and age^2^) [23,24]. The results of this variance component analysis (summarized under the headings Effect of Covariates and Basic Model in Tables 1 and 2) were consistent with preliminary analyses using mid-parent regression [25] and untransformed data (unpublished data). Further details, including estimates of individual variance components and likelihoods for each model are available online (http://www.sph.umich.edu/csg/chen/public/sardinia). When sex was modeled as covariate, sex differences explained 6.7% of the variance on average for blood test results, 16.2% for anthropometric measures, 4.3% for cardiovascular traits, and 2.4% for personality traits. When age and age^2^ were modeled as covariates, age differences typically explained a smaller proportion of the variance for blood tests results (5.1%) and personality traits (5.6%) than for anthropometric measures (20.4%) and for cardiovascular traits (25.3%). On average, the interaction between age and sex explained only a further 0.4% of the variance for all traits, and explained less than 3.0% of the variance for any individual trait.

**Table 1:**
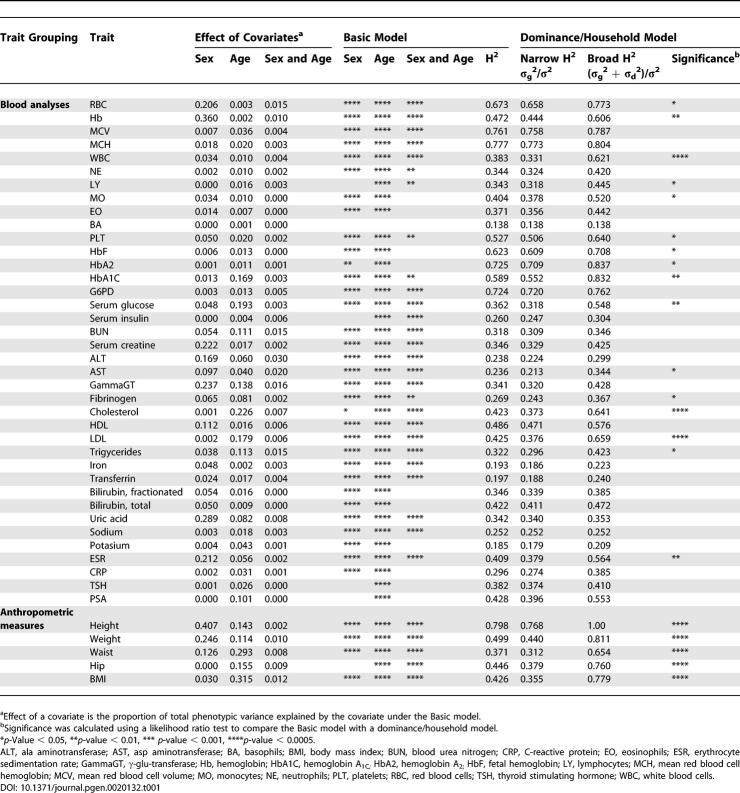
Heritability of Blood Phenotypes and Anthropometric Measures

**Table 2:**
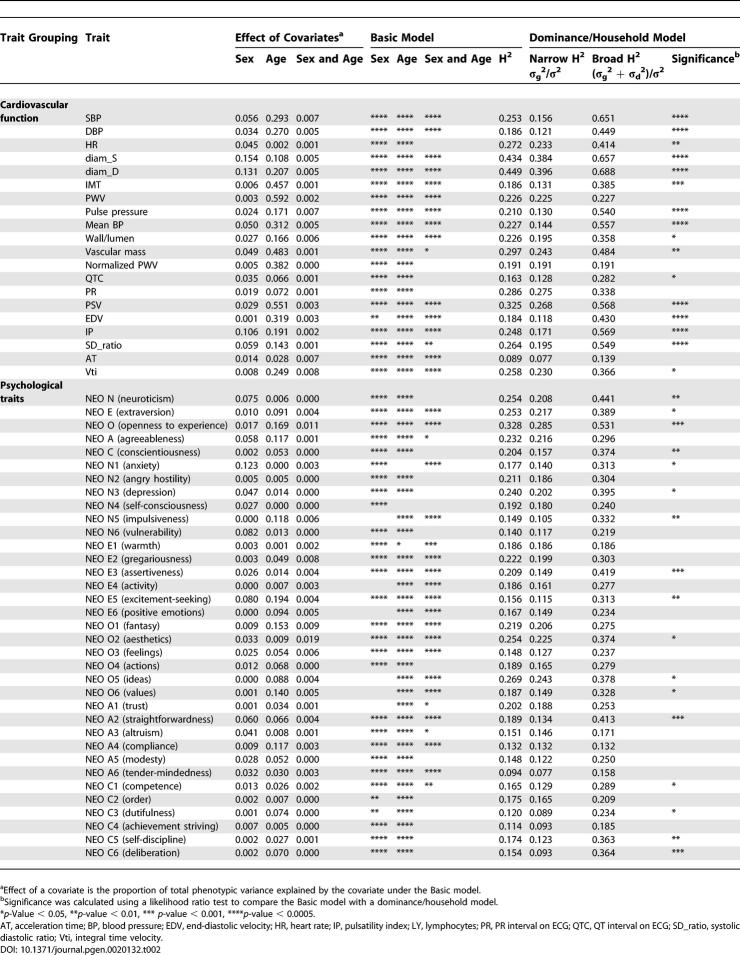
Heritability for Measures of Cardiovascular Function and Personality

A wide range of heritabilities was observed for each group of traits (Tables 1 and 2). After accounting for age and sex, heritability estimates ranged between 0.138 (for the fraction of basophils among white blood cells) to 0.777 (for the mean hemoglobin level in red blood cells, which is influenced by a common beta-thalassemia allele in Sardinia [8]) for the 34 blood tests; between 0.371 (for waist circumference) and 0.798 (for height) for five anthropometric traits; between 0.089 and 0.449 for 24 measures of cardiovascular structure/function, including values of 0.253, 0.186, 0.226, and 0.186 for the key variables of systolic blood pressure (SBP), diastolic blood pressure (DBP), pulse wave velocity (PWV), and intimal–medial thickness (IMT); and between 0.094 (for tender-mindedness, NEO A6) and 0.328 (for openness to experience, NEO O) for the 35 personality factors and facets. Notable results include substantial heritabilities for several risk factors for cardiovascular disease such as total cholesterol (0.423), HDL (0.486), LDL (0.425), and triglycerides (0.322), and the observation that the five personality factors typically show higher heritability (0.254 on average) than the 30 personality facets (0.177 on average).

All heritability estimates are statistically significant (*p* < 0.0005) and have a standard error of less than 0.023 (except for prostate-specific antigen [PSA], which was measured only in 2,604 males, and for which the standard error was 0.039, and thyroid stimulating hormone [TSH], which was measured in 3,461 individuals, and for which the standard error was 0.032).

### Models with Genetic Dominance or Shared Sibling Environment

We next proceeded to examine variance component models that allowed for either genetic dominance or shared sibling environment (Tables 1 and 2, last two columns). Although the two components of variance have different interpretations, they lead to equivalent predictions of trait variances and covariances for most relative pairs, and thus are hard to distinguish statistically in most datasets [26], including ours. We interpreted the estimate of heritability from a model allowing genetic dominance as a liberal estimate for the impact of genes on each trait, and the estimate from a model allowing instead for a contribution of shared sibling environment to the variance as a more conservative estimate. In most cases, the true heritability will be intermediate between these liberal and conservative estimates.

We detected a significant (*p* < 0.05) genetic dominance and/or shared sibling environment variance component for 53 of 98 traits. These included all anthropometric traits (five of five), most cardiovascular measures (16 of 20), and a substantial number of personality factors and facets (16 of 35), as well as blood test results (16 of 38). Including genetic dominance increases the average heritability from 0.403 to 0.496 for blood test results, from 0.508 to 0.799 for anthropometric measures, from 0.249 to 0.444 for measures of cardiovascular function, and from 0.188 to 0.299 for personality factors and facets.

### Heterogeneity in Variance Components, by Sex

Having evaluated the standard variance component models and estimated a heritable component for each trait, we proceeded to evaluate the evidence for heterogeneity in genetic and environmental sources of variance in males and females. We considered a series of models with heterogeneity in variance components (heterogeneity in environmental variance only, heterogeneity in genetic variance only, heterogeneity in both variance components, and a model in which the genetic and environmental variances differed between males and females by a constant factor). We also considered models that included an X-linked or mitochondrial variance component (because those types of non-autosomal inheritance also induce sex-dependent differences in covariances between relatives). We selected the best-fitting model for each trait using the Bayesian information criteria (BIC) criterion (Materials and Methods). Four traits (cholesterol, LDL, G6PD, and erythrocyte sedimentation rate [ESR]) appeared to be influenced by a substantial X-linked component. In females, we estimate that X-linked genes account for 7.5% of the variance in normalized cholesterol values, 9.5% of the variance in normalized LDL levels, 22.8% of the variance in normalized G6PD levels, and 6.2% of the variance for normalized ESR. In males, these proportions are approximately doubled. For the remaining 94 traits, the BIC criterion selected a model with heterogeneity between the sexes for 40 traits (summarized in Table 3) and a model with no heterogeneity for 54 traits. We found no significant evidence for mitochondrial inheritance of any trait.

**Table 3:**
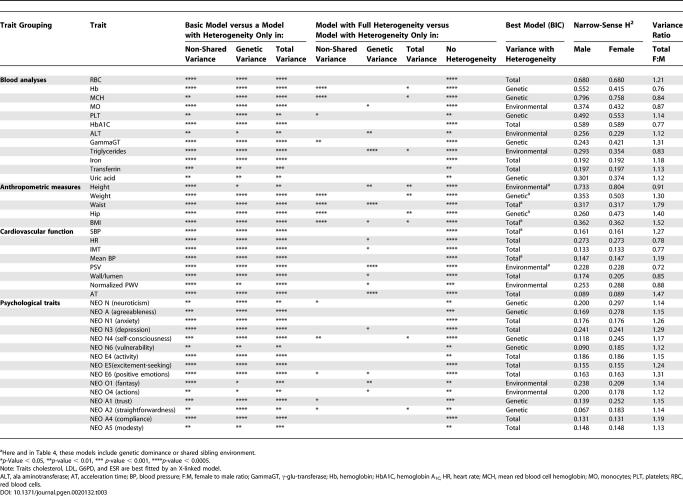
Model Comparisons between Males and Females

The 40 traits showing significant evidence for heterogeneity of variance components by sex included all five anthropometric traits and many of the blood test results (12 of 34), cardiovascular traits (eight of 20), and personality traits (15 of 35). When heterogeneity was detected, the BIC criterion selected a model with heterogeneity in environmental variances for eight traits (the environmental variance was larger among males in five cases); with heterogeneity in genetic variances for 13 traits (the genetic variance was larger among females in 11 cases); and a model with heterogeneity in the total variance for the remaining 19 traits (in these cases, the environmental and genetic variances differed by a constant factor between males and females, and the total variance was estimated to be larger among females in 15 cases). Interestingly, the biggest differences were observed for body weight (estimated heritability of approximately 50% in females, but approximately 35% in males), hip circumference (heritability of approximately 48% in females, but approximately 27% in males) and γ-glu-transferase levels (heritability of 42% in females, but 24% in males). The A (agreeableness), N (neuroticism), and E (extraversion) personality factors and four facets showed approximately 10% higher heritability in females.

Table 3 also provides a note of caution for our results. The three columns under the Basic Model header summarize comparisons of the base model with each of the heterogeneity models using a likelihood ratio test. For the tabulated traits, all heterogeneity models reject the base model (number of asterisks represents the *p*-value at which base model is rejected). In contrast, the next four columns, which compare a model with heterogeneity in both genetic and environmental variances to more parsimonious models, show that, quite often, multiple models with intermediate levels of heterogeneity give a similar fit to the most general heterogeneity model considered. For example, for the cardiovascular trait peak systolic velocity (PSV), both models with heterogeneity only in environmental variance and models with heterogeneity in the total variance fit as well as the model with full heterogeneity (*p* > 0.05, indicating no significant degradation in fit when using the parsimonious models). Thus, there was clear evidence for heterogeneity in variance components by sex, but it was difficult to decide whether the heterogeneity was due to genes, environment, or both.

### Heterogeneity in Variance Components, by Age

To look for heterogeneity in variance components by age, we divided individuals into two groups. The “younger” group included individuals less than 42 y of age (the median age in our sample), whereas the “older” group included individuals 42 y of age and older. We found significant evidence for heterogeneity in variance components by age in 62 of the 98 traits examined (the results are summarized in Table 4). This included a majority of traits in all categories, including anthropometric traits (three of five), blood test results (24 of 38), cardiovascular traits (13 of 20), and personality factors and facets (22 of 35). Again, we considered a series of intermediate models, including only heterogeneity in environmental or genetic variance components, or in which variance components differed by a constant factor between the young and old, and used the BIC to select the best-fitting model. For 26 traits, a model in which only the environmental variance differed between young and old was selected, and for 20 of these traits, environmental variance was greater among older individuals (so that heritability was lower). Heritability was higher in older individuals for IMT and five personality traits.

**Table 4:**
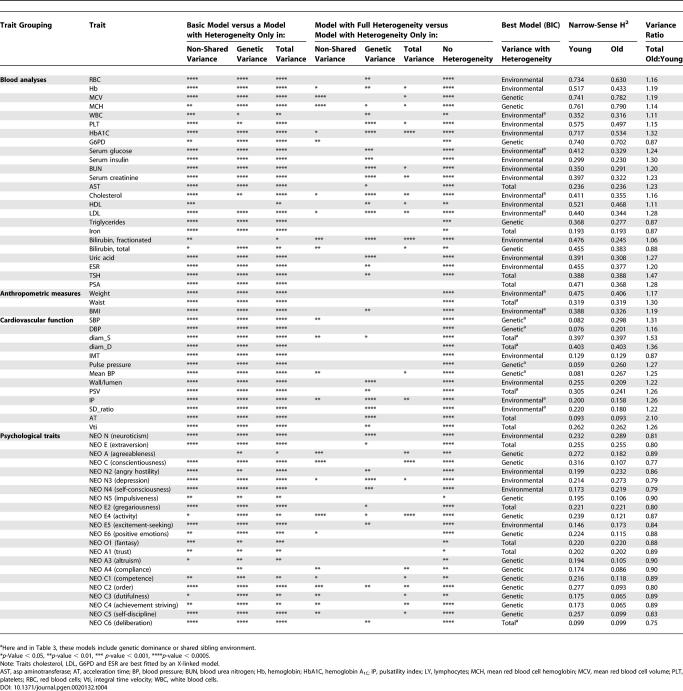
Model Comparisons between Young and Old

For 21 traits, a model in which only genetic variance differed between the young and old was selected, and heritability was higher in the young for 15 traits (12 personality traits and three blood test results). It is noteworthy that the six traits more heritable in the old included several blood pressure–related traits (SBP, DBP, mean blood pressure, and pulse pressure). For these cardiovascular traits, heritability increased an average of 18% among older individuals, from approximately 8% for younger individuals to approximately 26% in older individuals. For 15 other traits, a model in which heritabilities between the young and old differed by a constant factor provided the best fit to the data, whereas for one trait (fractionated bilirubin), both environmental and genetic variance components appeared to differ between the young and old.

### Bivariate Analysis

We calculated genetic correlation coefficients for all pairings of 93 traits (including the 38 blood phenotypes, five anthropometric measures, 20 cardiovascular traits, and 30 facets of personality, but excluding the five factors of personality, which are derived from the 30 facets). This corresponds to a total of 8,556 genetic correlation coefficients, of which 118 coefficients were greater than 0.50. In contrast, only 36 of the overall correlation coefficients were greater than 0.50. A full matrix of pairwise correlation coefficients is available http://www.sph.umich.edu/csg/chen/public/sardinia).

**Figure 4:**
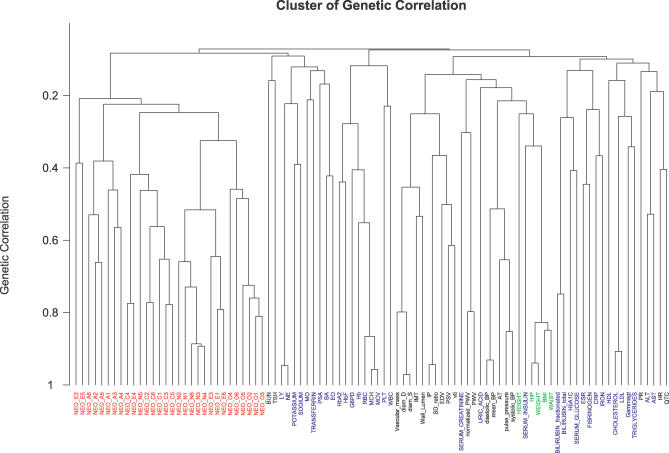
Clustering of Genetic Correlations The 98 quantative traits are classified into clusters inferred from genetic correlations between any two traits, with an “average” distance measure used in the clustering algorithm. Classes of traits are color-coded as personality (red), serum composition (blue), cardiovascular (black), and anthropometric (green). Overlap of the apparent genetic contribution to variance is indicated on the ordinate, with larger overlaps towards the bottom. Eighteen values exceed 50% overlap (see text).

We identified 18 clusters of traits with a genetic correlation greater than 0.50 (Table S2). To summarize the full pairwise correlation matrix, we used a hierarchical clustering approach that successively groups traits with large genetic correlations (see Figure 4). In the figure, traits connected by short branches share more of their genetic correlation, whereas traits that join up only near the root of the tree have only a small genetic correlation. Some of the clusters occur because traits are related by definition (for example, pulse pressure and SBP), or by physiology (for example, diastolic diameter [diam_D] and systolic diameter [diam_S], and IMT and wall lumen). Other clusters are quite interesting. For example, hip circumference, waist circumference, body mass index (BMI), and weight all cluster close together and near insulin levels. These traits are all related to the metabolic syndrome [27], and the result supports a genetic underpinning for the syndrome. As another example, the clustering of facets for the NEO O, NEO N, NEO C, and NEO A factors reinforces the structure of the five-factor personality model. Other results are more unexpected. For example, the personality facet NEO E4 (activity) clusters closer to components of NEO C (conscientiousness) than it does to other facets of NEO E. To further investigate the genetic relationship between different personality facets, we also carried out a factor analysis of genetic correlations (Table S3). This factor analysis confirms that the genetic structure of personality replicates its phenotypic structure quite well, but again places NEO E4 closer to components of NEO C.

We looked specifically for a genetic link between personality traits and cardiovascular disease [22]. Hostility, depression, anger, and anxiety have been associated with cardiovascular risk factors, including arterial stiffness and thickness (see [28] and references therein), and are independent predictors of incident cardiovascular disease and mortality [29]. Several mechanistic links have been proposed to explain the relationship between personality traits and cardiovascular diseases and outcomes [30]. However, the basis for the association has been conjectural. We find no substantive sharing of a genetic basis for cardiovascular traits and any psychological traits. For example, genetic correlation between N2 (hostility and anger) or A4 (low compliance/aggression) and IMT, PWV, SBP, DBP, or heart rate was not significantly different from zero.

## Discussion

The cohort of Sardinians described here provided us with a valuable opportunity to investigate the heritability of multiple traits simultaneously. For some traits, the size of our cohort exceeds the total number of individuals examined in all previously published studies of their heritability. The large size of the cohort and the diversity of the relationships sampled enabled us not only to consider the overall heritability of each trait, but also to investigate the possibility of heterogeneity in genetic effects by age or sex, as well as the evidence for shared genetic determinants between different traits. To facilitate downstream studies, complete results of all our analyses (including likelihoods and parameter estimates for each model fitted) are available online (http://www.sph.umich.edu/csg/chen/public/sardinia).

Overall, we estimated heritabilities of approximately 0.40 on average for individual blood test results, approximately 0.51 for anthropometric measures, approximately 0.25 for measures of cardiovascular function, and approximately 0.19 for personality factors and facets. In general, our results appear to be consistent with previous studies (see, for example, [31–34]), and particularly with previous studies based on extended pedigrees, (e.g., in the Hutterites [35] and another Sardinian village [36]). Our estimates of heritability are smaller than in previous studies of twins and siblings, both for cardiovascular traits [37,38] and for personality traits [39–43]. Extended pedigree samples such as ours allow specific assessment of narrow heritability potentially, and it is possible that non-additive effects inflated estimates of heritability in studies of twins and small families [44,45]. In our cohort, four of five components of the five-factor model (NEO N, E, O, and C) and most cardiovascular traits showed evidence for genetic dominance. Our broad estimates of heritability, which allow for genetic dominance, are more similar to results in studies of twins and siblings.

Nearly all traits showed highly significant evidence (*p* < 0.0005) for differences in trait means between the sexes (75 of 98 traits) and across age bands (91 of 98 traits). The evidence that sex and age play a key role in determining quantitative trait variation motivated us to investigate whether estimates of variance components (and therefore heritabilities) differed between the sexes, or between young and old. We found significant differences in variance components by sex for 40 traits (consistent with a recently published study of 17 quantitative traits in the Hutterites [46]). Not surprisingly, evidence for heterogeneity was found for the five anthropometric traits (height, weight, BMI, waist, and hip circumference) for which sexual dimorphism is obvious. More interestingly, we observed that, when there were differences in heritability by sex (21 traits), heritability was generally larger among females (in 16 traits). The remaining 19 traits showed heterogeneity in the total variance, but similar ratios of genetic and environmental variances within each sex. The differences were sometimes dramatic: weight had heritability of approximately 50% among females, but only approximately 35% among males; and neuroticism (NEO N), agreeableness (NEO A), and extroversion (NEO E) all had heritabilities of approximately 30% among females, but only approximately 20% among males.

Similarly, we found age differences in variance components for 62 traits. In some cases, these differences affected the genetic variance; in other cases, they affected environmental variances; and in still other cases, they affected the total variance. In the majority of cases in which we saw a difference in heritabilities between the young and old, we observed higher heritabilities among younger individuals. The trend likely reflects an expected increase of environmental insults with age [47]. Nevertheless, important exceptions were present, including traits such as SBP, DBP, mean blood pressure, and pulse pressure, whose heritability increased an average of 18% among older individuals. Interestingly, some measures of vascular structure and function (such as PWV, which reflects autonomous stiffening of the arterial substrate) that change markedly with age, showed no significant differences in variance components between young and old individuals. Taken together, these results suggest that it will often be fruitful to examine genetic effects separately by sex and age groups. In some instances, our results may allow investigators to focus molecular studies on groups that show higher heritability.

Although our previous discussion focused on total heritability, our analysis also allowed us to examine the effects of X-linked loci. We found a substantial X-linked component influencing G6PD levels—a result that was expected, because mutations in the G6PD gene *(Xq28)* are relatively common in Sardinia where they reduce G6PD levels and protect against malaria [48]. G6PD levels also exhibited a substantial autosomal component, supporting the presence of other regulatory loci that may influence malaria susceptibility and resistance through regulation of G6PD levels. In addition, we found a substantial X-linked variance component for three other quantitative traits (cholesterol, LDL, and ESR). In some of these cases, an X-linked component has been mapped (for an example of an X-linked gene influencing cholesterol levels, see [49]), and in other cases, our results may facilitate gene mapping and identification.

Although the relationship between variance components and the effects of age and sex remains conjectural, and the particular source of the heterogeneity was sometimes hard to distinguish, Tables 3 and 4 clearly show that heterogeneity in variances is too great to be ignored in analyses of many traits: for the traits listed, models without heterogeneity were always rejected when compared to models with heterogeneity. Thus, modeling the variance heterogeneity between different groups or stratifying analysis by age or sex could be valuable in molecular studies. In addition, it may be desirable to focus sample collection and analysis in genetic studies on the most informative individuals (for example, our results show traits such as blood pressure have very low heritability in individuals less than 40 y of age and may be more fruitfully studied in older individuals).

We found that the correlation of genetic variance components across age groups and across sexes did not significantly deviate from 1.0 and thus, despite evidence for heterogeneity, our results do not suggest that different genes determine heritability in males and females, or in the young and old. Instead, we infer that, at any age, the alleles involved consistently increase (or decrease) values of a particular trait in relation to the age-specific population mean. If the cumulative effects of these alleles become functionally severe only at older ages, when reproductive life is generally over, deleterious alleles may still reach substantial frequencies in the population.

Further analyses can also benefit from the apparent overlap in the genetic determinants of multiple traits. For example, our observations can guide downstream multivariate analysis as well as the construction of composite traits. Combining traits with a shared genetic component can result in composite traits with higher heritability than their component phenotypes [50], increasing power. Combining other trait groupings will likely be less helpful. In some cases, observed overlaps are persuasive, such as, for example, overlap in the genetic components related to the metabolic syndrome. For personality traits, our analyses imply that the facets defining each factor share major genetic determinants, and that the phenotypic organization of the facets into higher order factors is genetically rooted, an inference supported by direct analysis of genetic factor structure (see Table S3). Interestingly, we found no evidence for the simple notion that a shared genetic determinant can be responsible for both personality and cardiovascular traits. To account for the association, more complex hypotheses must thus be entertained, possibly involving shared environmental factors or gene–environment interactions.

In ongoing studies, we plan to refine heritability estimates for traits sensitive to major environmental factors, extending the analyses by taking into account recorded information about blood pressure medicines, smoking, and alcohol consumption. We are also attempting to assess possible ascertainment bias at older ages, resulting from the differential death rates among individuals with constitutions associated with premature death (for example, individuals with high blood pressure). In one preliminary approach, we limited ascertainment bias by excluding those over 60 y of age from the analysis (also eliminating most individuals taking blood pressure medication or affected by cardiovascular disease). Nevertheless, even with this truncated sample, estimated heritabilities in young and old individuals follow similar trends to those reported here, and our qualitative conclusions are not affected (work in progress). Ultimately, decisive analyses should be possible based on further longitudinal study of the current cohort.

Overall, the cohort provides a high-yield setting for identifying traits controlling variation in medically important quantitative traits in humans. We originally planned to focus genetic analyses on individuals with extreme values for a few cardiovascular and personality traits. However, technological advances have greatly facilitated large-scale genotyping, and we now expect that a single genome scan can be completed including all the individuals in our cohort. This will allow analysis for multiple traits, and these heritability analyses results suggest that the cohort will provide a valuable resource for gene mapping. Standard power calculations suggest that a linkage scan of these samples should yield expected LOD (logarithm of the odds ratio) scores of 3 or greater for loci explaining more than 8% of the variation in 96 of 98 traits (all but PSA and TSH; W.-M. Chen, unpublished data), whereas a genome-wide association scan could identify common alleles explaining as little as 1% of the variation. Simultaneous genetic analysis of multiple traits in a single cohort will necessarily involve a substantial amount of multiple testing, but careful evaluation of false discovery rates [51] (for example, by comparing the number of LOD scores exceeding a particular threshold with that expected under the null), should help interpret the results of our gene-mapping analyses. Because the range of human variation largely extends across human populations [52], our results should be relevant to genetic studies not just in Sardinia, but also in other populations.

## Materials and Methods

### Population sampling.

Recruitment focused on Lanusei—the largest town in Ogliastra, the location of its only hospital, and site of the local bishopric—and the neighboring towns of Ilbono, Arzana, and Elini. To achieve our goal of recruiting more than 6,000 individuals from the region, the project was advertised through provincial, religious, and municipal authorities, in local television, newspaper, and radio messages, through local physicians, and by mailings and phone calls. Only individuals more than 13 y of age were eligible to participate in the study.

A clinic was established in a quiet but easily accessible sector of Lanusei. Each subject came to the clinic before breakfast, signed consent forms, and gave a sample of fasting blood. Later in the day, each subject returned for a full 2-h evaluation, including blood pressure and anthropometric measurements, cardiovascular assessments and personality testing, and a medical history interview. Two teams of six staff, all Sardinian, worked in parallel so that up to 60 subjects could be examined each week. Each team included: a physician, responsible for the medical history and physical examination; another physician and a technician, responsible for measurements of arterial stiffness and thickness; a tester for the psychological inventory; and, finally, a phlebotomist and a technician responsible for collecting and fractionating blood. Additional backup staff helped in data handling and transfer.

### Institutional review board approval.

The study, including the protocols for subject recruitment and assessment; the Informed Consent for participants (and Assent Forms for those 14–18 y); and the overall analysis plan was reviewed and approved by institutional review boards for the Istituto di Neurogenetica e Neurofarmacologia (INN; Cagliari, Italy), for the MedStar Research Institute (responsible for intramural research at the National Institutes of Aging, Baltimore, Maryland, United States) and for the University of Michigan (Ann Arbor, Michigan, United States).

### Psychological phenotypes.

Personality phenotypes were assessed with the Revised NEO Personality Inventory (NEO-PI-R [53]), a questionnaire consisting of 240 items answered on a five-point Likert scale ranging from *strongly disagree* to *strongly agree*. The NEO-PI-R characterizes consistent patterns of thought, feeling, and action for each individual. Five major factors [neuroticism (N), extraversion (E), openness to experience (O), agreeableness (A), and conscientiousness (C)], each of which is a composite of six facets of personality, are measured. The NEO-PI-R provides a comprehensive and detailed assessment of normal adult personality in terms of emotional, interpersonal, experiential, attitudinal, and motivational styles. The inventory has a robust factor structure that has been replicated in Italy [54] and in more than 50 cultures [55]. Scales have shown longitudinal stability, cross-observer agreement, and convergent and discriminant validity in a large body of studies [21]. Two trained Sardinian psychologists administered the tests orally to participants unable to fill out the questionnaire.

### Blood composition.

From each participant screened, 25 ml of blood was drawn and fractionated to provide serum, EDTA-plasma, heparin-plasma, white blood cells, and red blood cells. Clinical laboratories in Sardinia provided blood cell counts and applied a standard battery of blood tests for the measurement of electrolytes, renal function, liver function, thyroid function, and iron metabolism. Given our interest in cardiovascular risk factors, fasting lipid profiles, markers of insulin resistance (glucose, insulin, and hemoglobin A_1C_), and ESR were also measured. C-reactive protein (CRP) was assessed using the standard low-sensitivity assay [56].

### Cardiovascular profile.

Blood pressure was measured with a mercury sphygmomanometer. Measurements were taken in the morning, after a light breakfast, and after a 5-min quiet resting period, with subjects in the seated position. The SBP and DBP used here are the average of the second and third measurements from the right arm. Pulse pressure (PP) was calculated as (PP = SBP − DBP) and mean blood pressure (MBP) as (MBP = DBP + PP/3). Standard 12-lead electrocardiography was performed on all participants, from which the PR interval and the QT interval corrected for heart rate (QTC) were measured.

Participants also underwent non-invasive assessments of arterial structure and function that are increasingly recognized as potent predictors of adverse cardiovascular outcomes [15,57,58]. Carotid–femoral PWV, an index of central arterial stiffness, was measured with the help of transcutaneous Doppler ultrasonography [59]. Carotid ultrasonography was performed for the measurement of arterial diam_S and diam_D, and IMT; from these variables, vascular mass and the ratio of wall thickness to lumen diameter were calculated. PWV, IMT, and carotid diameter measurements were performed off-line by a single observer (A. Scuteri) who was blinded to the identity of participants.

During the sonographic evaluation of the common carotid artery, Doppler studies allowed the measurement of PSV, end-diastolic velocity (EDV), pulsatility index (IP), systolic–diastolic ratio (SD_ratio), and acceleration time (AT).

### Additional traits and details.

In addition to personality traits, cardiovascular measures, and blood composition, we also considered four anthropometric traits recorded during physical examination of each subject (height, weight, and waist and hip circumference) and one derived quantity (the BMI, kg/m^2^). For conciseness, additional details of how individual phenotypes, including cardiovascular measures, were collected are supplied as Protocol S1.

### Quality assessment.

Quality assessment of the data was carried out using PEDSTATS [60], and in-house SAS (SAS Institute, Cary, North Carolina, United States) and R (The R Project for Statistical Computing [http://www.r-project.org]) scripts. These tools allowed us to check that pedigrees were self-consistent, and to identify univariate and multivariate outliers. Outliers and other unusual trait values were inspected against the original records whenever possible.

### Variance components analysis.

To utilize fully the information in our cohort, and to accommodate covariate effects, we estimated heritabilities using a variance components model [23,24]. This model can accommodate pedigrees of any configuration and is well suited to the analysis of extended pedigrees. We included age, age^2^, sex, and the two corresponding interaction terms as covariates in all analyses. We considered a series of models for each trait, and these are detailed below, together with details of transformations we applied to each trait to guard against the possibility of statistical artifacts induced by outliers or non-normal marginal distributions.

### Quantile normalization.

Variance components analyses are sensitive to outliers, kurtosis, and skewness in the trait distribution. Quantile normalization provides a practical way to deal with these problems in the context of gene mapping and, specifically, variance component analyses [61–63]. For traits that are approximately normally distributed, normalization has minimal impact on results. For other traits, normalization will not induce correlations between relatives not present in the original data and thus should not lead to erroneous inference of a heritable component for variation. To carry out quantile normalization, we first ranked the observations and then matched the percentile of each observation to the corresponding percentile in a standard normal distribution. Using the resulting percentiles, we replaced each observation with the corresponding *z*-score from the standard normal distribution. When ties were present, percentiles were averaged across all ties.

### Base polygenic model.

We considered a base model in which variance is partitioned into a polygenic component σ_g_^2^ and an environmental component σ_e_^2^. As usual, the environmental component is unique to each individual, whereas the polygenic component is shared between individuals in proportion to their kinship coefficient. Thus, if *Y_i_* is the observed trait measurement for individual *i,* we define its variance as *Var(Y_i_) =* σ_g_^2^ + σ_e_^2^ and the covariance between measurements for a pair of individuals *i* and *j* with kinship *ϕ_ij_* as *Cov(Y_i_, Y_j_) =* 2 *ϕ_ij_* σ_g_^2^.

After fitting this base model, we considered refined models including additional variance components to model genetic dominance, σ_d_^2^, or the effects of shared sibling environment, σ_s_^2^. To model genetic dominance, we let *Δ_ij_* denote the probability that individuals *i* and *j* share two alleles identical by descent (IBD), based on their reported relationship (as usual, this quantity was calculated using generalized kinship coefficients [64]). Then we modeled the variance of each trait measurement as *Var(Y_i_) =* σ_g_^2^ + σ_e_^2^ + σ_d_^2^ and the covariance between measurements for a pair of individuals as *Cov(Y_i_, Y_j_) =* 2 *ϕ_ij_* σ_g_^2^ + *Δ_ij_* σ_d_^2^. To model the effects of shared sibling environment, we let *I_sib(i,j)_* be an indicator variable with value 1 when individuals *i* and *j* are full sibs, and value 0 otherwise. Then we let*Var(Y_i_) =* σ_g_^2^ + σ_e_^2^ + σ_s_^2^ and *Cov(Y_i_, Y_j_) =* 2 *ϕ_ij_* σ_g_^2^ + *I_sib(i,j)_* σ_s_^2^. In most datasets, including our own, the two models cannot be distinguished statistically [26]. In fact, because *Δ_ij_ = 0.25* and *I_sib(i,j)_ = 1* when *i* and *j* are full siblings, and *Δ_ij_* = *I_sib(i,j)_=* 0 for *nearly all* other pairs of individuals, it is simple to show that the two models lead to identical predictions of variances and covariances when we set σ_d_^2^ = 4 σ_s_^2^ and adjust σ_e_^2^ appropriately. Although our dataset does not allow us to distinguish between models with only genetic dominance (σ_d_^2^ > 0, σ_s_^2^ = 0), models with only shared environment (σ_d_^2^ = 0, σ_s_^2^ > 0), and other intermediate models (σ_d_^2^ > 0, σ_s_^2^ > 0), comparisons of parameter estimates from these models are informative. In the model with genetic dominance, the quantity H^2^ = (σ_d_^2^ + σ_g_^2^)/(σ_d_^2^ + σ_g_^2^ + σ_e_^2^) provides a liberal estimate of the overall impact of genes on the phenotype at hand, whereas in the model attributing any excess similarity between siblings to shared environment, the quantity h^2^ = σ_g_^2^/(σ_s_^2^ + σ_g_^2^ + σ_e_^2^) provides a very conservative estimate of the overall impact of genes. Whenever there was significant evidence (*p* < 0.001) for shared sibling environment or genetic dominance, genetic dominance was included in the heterogeneity analyses described below.

### Models with heterogeneity between the sexes.

To model heterogeneity, we evaluated models in which separate variance components were fitted for males, σ_g,male_^2^ and σ_e,male_^2^, and females, σ_g,female_^2^ and σ_e,female_^2^. The variances for each trait measurement and the covariances for trait-measurements between individuals of the same sex follow naturally from the formulae given in the section describing the Base Polygenic Model (above). When individuals *i* and *j* were of opposite sexes, we set the covariance to *Cov(Y_i_, Y_j_) =* 2 *ϕ_ij_* 
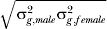
 . When there was evidence for heterogeneity, we proceeded to consider a series of intermediate models in which heterogeneity was allowed only for environmental effects (i.e., where σ_g,male_^2^ = σ_g,female_^2^), only for genetic effects (i.e., where σ_e,male_^2^ = σ_e,female_^2^) or where variability increased uniformly for both genetic and environmental factors (σ_g,male_^2^ = *k* σ_g,female_^2^ and σ_e,male_^2^ = *k* σ_e,female_^2^, with *k ≠ 1*).

Setting *Cov(Y_i_, Y_j_) =* 2 *ϕ_ij_* 
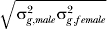
 for opposite sex individuals assumes that the same genes influence phenotypes for males and females. We also evaluated a model in which the covariance for individuals of opposite sexes was *Cov(Y_i_, Y_j_) =* 2 *ϕ_ij_ ρ_sex_* 
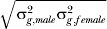
 , but we found no cases where this model (with *−1 < ρ_sex_ < 1*) resulted in a significant improvement in the likelihood. Thus, our data provide no evidence that different genes contribute to genetic variation in males and females, but rather that the same genes make different contributions in each sex.

### Mitochondrial and X-linked inheritance.

We also considered the possibility of X-linked or mitochondrial inheritance. X-linked inheritance can produce differences in the total variance between males and females, and either of these phenomena can generate sex-dependent covariances between relatives (for example, they can lead to differences between mother–daughter correlations and father–son correlations). We compared models with heterogeneity to models with an X-linked variance component, σ_x_^2^, and to models with a mitochondrial genetic variance component, σ_m_^2^. In models with an X-linked variance component, *Var(Y_i_) =* σ_g_^2^ + σ_e_^2^ + 2 *ϕ_ii_^(X)^* σ_x_^2^ and *Cov(Y_i_, Y_j_) =* 2 *ϕ_ij_* σ_g_^2^ + 2 *ϕ_ij_^(X)^* σ_x_^2^, in which calculation of the kinship coefficient for X-linked genes is detailed at the end of the Materials and Methods section. In models with a mitochondrial variance component, *Var(Y_i_) =* σ_g_^2^ + σ_e_^2^ + σ_m_^2^ and *Cov(Y_i_, Y_j_) =* 2 *ϕ_ij_* σ_g_^2^ + *M(i,j)* σ_m_^2^. The indicator function *M(i,j)* takes a value = 1 if individuals *i* and *j* are related through their maternal lineages, and 0 otherwise.

### Models with heterogeneity between young and old.

Similarly to our analysis with heterogeneity by sex, we defined separate variance components for individuals whose age was greater or equal than the sample median (42 y of age), σ_g,old_^2^ and σ_e,old_^2^, and for individuals whose age was below the sample median, σ_g,young_^2^ and σ_e,young_^2^. When there was evidence for heterogeneity, we considered intermediate models in which heterogeneity was allowed only for environmental effects (i.e., where σ_g,young_^2^ = σ_g,old_^2^), or only for genetic effects (i.e., where σ_e,young_^2^ = σ_e,old_^2^), or in which the total variance for both genetic and environmental factors changed by a shared factor (σ_g,young_^2^ = *k* σ_g,old_^2^ and σ_e,young_^2^ = *k* σ_e,old_^2^, with *k ≠ 1*).

### Bivariate trait analyses.

To investigate the origin of correlations between each pair of traits *Y* and *Z,* we portioned the bivariate correlation into an environmental correlation, *ρ_e(Y,Z)_,* and a genetic correlation, *ρ_g(Y,Z)_,* for each pair of traits. To do this, we fitted the base polygenic model to each pair of traits simultaneously, and set the cross-trait correlation to *Cov(Y_i_,Z_i_) = ρ_e(Y,Z)_* 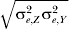 + *ρ_g(Y,Z)_* 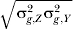 and the cross-trait, cross-individual correlation to *Cov(Y_i_,Z_j_) =* 2 *ϕ_ij_ ρ_g(Y,Z)_* 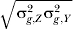 *.* To summarize the results, we defined |1 − *ρ_g(Y,Z)_|* as the distance between each pair of traits, and implemented a simple hierarchical clustering analysis [65], which successively connects the most similar traits using a greedy algorithm. This analysis was carried out in R, using the hclust() function and the “average” agglomeration method.

### Maximum likelihood.

All models were fitted by maximizing the standard multivariate normal likelihood [66], which depends on a linear model for expected values (incorporating a trait-specific mean as well as age, age^2^, and sex) and a model for variances and covariances. Likelihoods were maximized using a computationally efficient scoring method [64] implemented in the program POLY (freely available with source code from http://www.sph.umich.edu/csg/chen/poly). The program uses Generalized Estimating Equations estimates (GEE) [67] to select starting values for iterative likelihood maximization, implements various diagnostic techniques, and provides standard errors for all parameter estimates. The current version of the program can handle any non-inbred pedigree. For a subset of the results reported here, estimates were compared with those obtained from SOLAR [68] and QTDT [69], with identical results. We used a likelihood ratio test to compare nested models. To compare non-nested models, we used BIC [70].

### Kinship coefficient for X-linked loci.

Recall that the kinship coefficient is the probability that two identical alleles will be sampled from a pair of individuals when we select one allele at random from each. The self-kinship coefficient is the probability that two alleles sampled from one individual, with replacement, are identical. We used a recursive formulation to estimate kinship coefficients for X-linked genes, analogous to the conventional approach described in Lange [64] for autosomal genes. First, we ordered all individuals in a pedigree such that for any two individuals *i* and *j, i > j* implies that *i* is not an ancestor of *j* (any ordering where ancestors precede their descendants is suitable).

Then, we defined the kinship coefficient for X-linked genes, *ϕ_ij_^(X)^_,_* as follows:

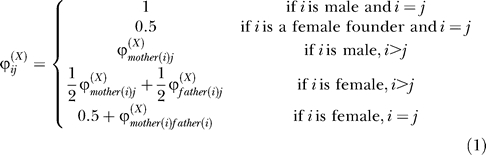



Although this definition only covers the situation in which *i* ≥ *j,* it can be used to estimate any kinship coefficient because *ϕ_ij_^(X)^* = *ϕ_ji_^(X)^*. The definition reflects the fact that males carry only one allele for X-linked genes, inherited from their mother. Females carry two alleles, one inherited from each parent. The functions *mother(i)* and *father(i)* return indexes for the parents of *i*.

## Supporting Information

Protocol S1 Supplementary Methodology: Protocol Details for Measuring Cardiovascular Traits This section provides a detailed protocol for the assessment of cardiovascular traits.
(18 KB PDF)

Table S1 Detailed Descriptive Statistics for 98 Traits This table includes trait means and variances. Trait means are stratified by sex and into four age bands.
(37 KB PDF)

Table S2 Clusters of Traits for Which Genetic Correlation Is More Than 0.5 Highlights subsets of traits identified in the clustering analysis, for which the genetic correlation exceeds 0.5.
(7 KB PDF)

Table S3 Genetic Factor Structure of Personality Traits The table presents Procrustes-rotated principal components from the genetic correlations among the 30 facets of the NEO-PI-R, targeted to the American normative factor structure.
(11 KB PDF)

## Abbreviations

BIC: Bayesian information criteria
BMI: body mass index
DBP: diastolic blood pressure
diam_D: diastolic diameter
diam_S: systolic diameter
ESR: erythrocyte sedimentation rate
HDL: high-density lipoprotein
IMT: intimal–medial thickness
LDL: low-density lipoprotein
PSA: prostate-specific antigen
PSV: peak systolic velocity
PWV: pulse wave velocity
SBP: systolic blood pressure
TSH: thyroid stimulating hormone
