# Supplementary material for: Heritability of Cardiovascular and Personality Traits in 6,148 Sardinians
Source: PLoS Genet. 2006 Aug 25;2(8):e132. doi: 10.1371/journal.pgen.0020132 (PMC1557782; doi:10.1371/journal.pgen.0020132)
Supplement: Protocol S1 — This section provides a detailed protocol for the assessment of cardiovascular traits. (18 KB PDF) [file pgen.0020132.sd001.pdf]

METHODOLOGY

Serum component values. Standard automated clinical chemistry was carried out on a Bayer Express 500 Plus instrument for all tests except sodium and potassium (Biotecnica Instruments, SpA, Flame Photometer BT634); erythrocyte sedimentation rate (Terumo Medical Corporation Sedimentation Tube Rack); PSA and TSH (DPC Immulite 1000, Diamond Diagnostics); and fibrinogen (Helena Biosciences COA Data 2001). The low-sensitivity CRP assay from the Bayer Express 500 Plus is entered as a trait, but is too insensitive to quantify CRP levels in healthy cohorts, and will be repeated with a high sensitivity assay after second visits of participants.

Anthropometric measurements. Height was measured without shoes and weight was measured with light clothing, using a rigid height scale and an attached "mechanical" balance that was periodically calibrated. Body mass index (BMI) was calculated as weight in kilograms divided by the square of the height in meters. The waist was defined in a standard way as the minimal abdominal circumference between the lower edge of the rib cage and the iliac crests. The hip was defined as the maximal circumference around the gluteal muscles below the iliac crests. The circumferences were obtained with a flexible plastic tape measure while maintaining close contact with skin and without compressing the underlying tissues. Subjects were in a standing position and breathing normally.

Cardiovascular measurements. To avoid interference, participants refrained from smoking for at least 3 hours before PWV and other vascular measurements.

Blood pressure. Blood pressure determinations were performed in the morning, after a light breakfast, with subjects in the seated position, and following a five minute quiet resting period. Blood pressure was measured in both arms with a standard mercury sphygmomanometer using an appropriately sized cuff. Measurements were rounded to the nearest even number (2 mm Hg interval). The blood pressure values used in this study are the average of the second and third measurements on the right arm. Values for systolic blood pressure (SBP) and diastolic blood pressure (DBP) were defined by Korotkoff phase I and V, respectively. Pulse pressure was computed as PP=(SBP-DBP); mean BP was computed as MBP= DBP +(PP / 3).

Intimal-medial thickness (IMT). IMT was measured by carotid ultrasonography as previously described (Scuteri et al., 2004). High-resolution B-mode carotid ultrasonography was performed by use of a linear-array 5- to 10-MHz transducer (Ultramark 9 HDI, Advanced Technology Laboratories, Inc). The subject lay in the supine position in a dark, quiet room. The right common carotid artery (CCA) was examined with the head tilted slightly upward in the midline position. The transducer was manipulated so that the near and far walls of the CCA were parallel to the transducer footprint and the lumen diameter was maximized in the longitudinal plane. A region 1.5 cm proximal to the carotid bifurcation was identified. The systolic and diastolic luminal diameters were measured visually with the help of EKG (in late-systole, i.e. the end of T wave, and in end-diastole, i.e. the peak of R wave, respectively). Luminal diameters were measured between both endothelial layers, perpendicular to the course of the vessel. The IMT of the far wall was evaluated as the distance between the luminal-intimal interface and the medial-adventitial interface. IMT was measured on the frozen frame of a suitable longitudinal image with the image magnified to achieve a higher resolution of detail by

dedicated software (HDI Lab, ATL, Phillips Medical Systems Inc.). Because the machine allows readings to only two figures, measurements fall into corresponding intervals; the population distribution showed essentially all values higher than 0.4 mm. All the measurements were performed by a single reader (A.S.). His intraclass correlation coefficient was 0.95.

QT interval. QT interval was measured with a caliper on a paper copy of the EKG by a single reader, a cardiologist (M.O.). The QTc interval was calculated as the QT interval corrected for heart rate.

Pulse Wave Velocity (PWV). PWV was derived from simultaneous recordings of arterial flow waves from the right common carotid artery and the right femoral artery as previously described (Vaitkevicius 1993). A minimum of 10 arterial flow waves from both sites were simultaneously recorded using nondirectional transcutaneous Doppler probes (model 810A, 9 to 10-Mhz probes, Parks Medical Electronics, Inc., Aloha, Or), and averaged using the QRS for synchronization. The foot of the flow, i.e. the point of systolic flow onset, was identified off-line by a custom-designed computer algorithm, and verified or manually adjusted by the reader after visual review. The time differential between the feet of simultaneously recorded carotid and femoral arterial flow waves was then measured. The distance traveled by the flow wave was measured with an external tape measure over the body surface, as the distance from the right carotid recording site to the manubrium subtracted from the distance from the manubrium to the right femoral arterial recording site. PWV was calculated as the distance traveled by the flow wave divided by the time differential. All arterial wave forms were evaluated by a single observer (A.S.) blinded with respect to the identity of data. His intraclass correlation coefficient is 0.80.

Quality control for cardiovascular measures. A single investigator (A.S.) reviewed all PWV waveforms and carotid ultrasound images throughout the study for quality control, and provided feedback to the testers as needed. Study personnel involved in performance of carotid ultrasonography and acquisition of PWV waveforms also underwent periodic retesting on a subgroup of participants to help ensure acceptable reproducibility.

Scuteri A, Najjar SS, Muller DC, Andres R, Hougaku, H, Metter EJ, Lakatta EG (2004) Metabolic syndrome amplifies the age-associated increases in vascular thickness and stiffness. J Am Coll Cardiol 43: 1388-1395.

Mackey RH, Sutton-Tyrrell K, Vaitkevicius PV, Sakkinen PA, Lyles MF, Spurgeon HA, Lakatta EG, Kuller LH (2002) Correlates of aortic stiffness in elderly individuals: a subgroup of the Cardiovascular Health Study. Am J Hypertens 15:16-23.
