# Supplementary material for: Heritability of Cardiovascular and Personality Traits in 6,148 Sardinians
Source: PLoS Genet. 2006 Aug 25;2(8):e132. doi: 10.1371/journal.pgen.0020132 (PMC1557782; doi:10.1371/journal.pgen.0020132)
Supplement: Table S1 — This table includes trait means and variances. Trait means are stratified by sex and into four age bands. (37 KB PDF) [file pgen.0020132.st001.pdf]

Supplementary Table 1: Summary Statistics for 98 quantitative traits

		Overall		Males				Females				Significant Variation	
**Age Band**				14-29 yrs	30 - 44 yrs	45 - 59 yrs	60-102 yrs	14-29 yrs	30 - 44 yrs	45 - 59 yrs	60-102 yrs	Age	Sex
**# individual¶**		6111	(43.6)	688.3	743.0	611.7	564.2	904.8	1049.6	851.3	698.2		
**Blood Test Analyses**	**Units**												
RBC	10^6/uL	4.8	(0.6)	5.3	5.2	5.1	4.9	4.6	4.6	4.7	4.7	****	****
Hb	g/dL	13.8	(1.5)	14.9	14.9	14.9	14.7	13.0	12.8	13.1	13.4	****	****
MCV (mean RBC vol)	fL	86.5	(9.3)	85.0	86.5	88.0	90.2	85.0	85.4	86.0	87.6	****	****
MCH (mean RBC Hb)	pg	28.8	(3.6)	28.6	29.1	29.4	30.1	28.3	28.4	28.5	29.0	****	****
WBC	10^3/uL	6.7	(1.7)	7.1	7.0	7.1	6.9	6.7	6.5	6.2	6.2	****	****
NE (neutrophils)	%	56.6	(8.7)	54.6	56.6	56.1	57.5	55.1	58.3	57.2	57.0	****	****
LY (lymphocytes)	%	34.5	(7.9)	35.8	34.3	34.3	32.4	36.7	33.7	34.2	34.2	****	
MO (monocytes)	%	5.9	(2.3)	6.6	6.0	6.3	6.7	5.6	5.3	5.6	5.8	****	****
EO (eosinophils)	%	2.6	(1.8)	2.7	2.7	2.9	3.0	2.3	2.3	2.6	2.6	****	****
BA (basophils)	%	0.4	(0.3)	0.4	0.3	0.3	0.3	0.4	0.4	0.4	0.4		**
PLT (platelets)	10^3/uL	243.0	(60.0)	234.0	231.0	227.0	220.0	263.0	258.0	256.0	237.0	****	****
HbF	%	0.4	(0.7)	0.4	0.3	0.3	0.3	0.5	0.5	0.4	0.4	****	****
HbA2	%	3.1	(1.0)	3.1	3.1	3.1	3.0	3.1	3.1	3.0	2.9	**	
HbA1C	%	5.4	(0.8)	5.2	5.3	5.8	5.9	5.1	5.2	5.5	5.9	****	****
G6PD	UI/dL	1.2	(0.4)	1.3	1.3	1.2	1.2	1.3	1.2	1.2	1.2	****	
SERUM GLUCOSE	mg/dL	90.1	(23.7)	84.7	89.8	100.0	104.0	79.0	82.9	90.4	100.0	****	****
SERUM INSULIN	micro/UmL	8.5	(8.1)	7.4	8.1	9.8	9.0	8.3	7.6	8.8	9.8	***	
BUN	mg/dL	35.0	(10.4)	34.8	37.6	37.5	40.9	28.4	31.5	34.8	39.9	****	****
SERUM CREATININE	mg/dL	0.8	(0.2)	0.9	0.9	0.9	1.0	0.7	0.7	0.7	0.8	****	****
ALT, ala aminotransferase	U/L	25.0	(23.1)	26.6	37.2	34.1	28.6	16.0	17.9	22.5	24.8	****	****
AST, asp aminotransferase	U/L	21.6	(14.6)	22.2	24.4	25.5	25.8	17.2	17.4	20.7	24.2	****	****
Gammagt, γ-glu-transferase	U/L	27.9	(39.9)	22.0	41.1	51.2	46.7	13.8	15.8	21.9	27.7	****	****
FIBRINOGEN	mg/dL	330.0	(67.4)	289.0	300.0	316.0	347.0	326.0	332.0	348.0	375.0	****	****
CHOLESTEROL	mg/dL	208.0	(42.2)	175.0	213.0	226.0	220.0	185.0	204.0	226.0	228.0	****	
HDL	mg/dL	64.1	(14.9)	55.6	57.1	59.7	62.6	66.3	68.3	69.7	69.0	****	****
LDL	mg/dL	127.0	(35.4)	105.0	134.0	141.0	136.0	106.0	121.0	139.0	140.0	****	**
TRIGLYCERIDES	mg/dL	88.1	(68.3)	72.5	111.0	127.0	106.0	61.8	69.9	84.8	95.4	****	****
IRON	microg/dL	87.7	(34.8)	94.1	98.3	96.4	95.2	80.2	80.1	81.1	85.7	*	****
TRANSFERRIN	mg/dL	307.0	(62.9)	301.0	293.0	297.0	292.0	326.0	325.0	308.0	297.0	****	****
BILIRUBIN, fractionated	mg/dL	0.1	(0.1)	0.1	0.1	0.1	0.2	0.1	0.1	0.1	0.1	****	****
BILIRUBIN, total	mg/dL	0.7	(0.4)	0.8	0.8	0.7	0.8	0.6	0.6	0.6	0.7	****	****
URIC ACID	mg/dL	4.3	(1.5)	4.8	5.1	5.4	5.7	3.2	3.3	3.8	4.4	****	****
SODIUM	mEq/L	143.0	(3.6)	142.0	143.0	143.0	143.0	142.0	142.0	143.0	143.0	****	****
POTASSIUM	mEq/L	4.4	(0.4)	4.3	4.4	4.5	4.6	4.3	4.4	4.5	4.5	****	****
ESR, erythrocyte sed. rate	mm/h	10.6	(8.2)	5.8	6.4	7.1	10.9	11.0	11.9	13.4	16.8	****	****
CRP, C-reactive protein	mg/dL	0.7	(1.1)	0.6	0.7	0.7	0.9	0.7	0.7	0.8	0.9	****	
TSH, thyroid stim. hormone	microU/L	1.9	(2.6)	4.2	2.5	1.0	2.0	1.8	1.9	1.9	1.7		
PSA		0.8	(1.1)	0.5	0.6	0.7	1.4					****	

		Overall		Males				Females				Significant Variation	
**Age Band**				14-29 yrs	30 - 44 yrs	45 - 59 yrs	60-102 yrs	14-29 yrs	30 - 44 yrs	45 - 59 yrs	60-102 yrs	Age	Sex
**# individual¶**		6111	(43.6)	688.3	743.0	611.7	564.2	904.8	1049.6	851.3	698.2		
**Anthropometric Measures**													
HEIGHT	cm	160.0	(9.1)	169.0	169.0	165.0	161.0	158.0	157.0	153.0	150.0	****	****
WEIGHT	kg	64.9	(13.3)	66.4	75.1	76.0	73.0	53.4	57.8	63.2	64.0	****	****
WAIST	cm	84.8	(13.1)	80.5	89.4	95.0	97.7	72.2	77.1	84.9	92.0	****	****
HIP	cm	97.6	(8.3)	93.8	98.0	98.9	99.0	93.3	96.3	101.0	102.0	****	*
BMI, body mass index	kg/m^2	25.3	(4.7)	23.1	26.1	27.8	28.2	21.3	23.5	26.8	28.5	****	****
**Cardiovascular Function**													
systolic BP	mmHg	126.0	(18.5)	121.0	125.0	135.0	144.0	111.0	114.0	129.0	140.0	****	****
diastolic BP	mmHg	77.0	(10.9)	71.0	79.2	84.9	83.9	68.4	72.8	80.0	81.7	****	****
HR (heart rate)	beats/min	67.1	(11.2)	64.8	63.8	65.7	64.2	70.1	68.7	68.5	68.8	*	****
diam_S	mm	5.9	(0.7)	6.0	6.2	6.3	6.6	5.5	5.5	5.7	6.2	****	****
diam_D	mm	5.4	(0.7)	5.3	5.6	5.8	6.2	4.9	5.1	5.2	5.7	****	****
IMT (intimal medial thickness)	mm	0.6	(0.1)	0.5	0.5	0.6	0.7	0.5	0.5	0.6	0.7	****	****
PWV (pulse wave velocity)	cm/s	671.0	(226.0)	513.0	623.0	741.0	925.0	495.0	589.0	721.0	923.0	****	****
Derived variables													
pulse pressure	mmHg	48.6	(13.0)	49.8	45.9	50.1	59.9	43.0	41.5	48.6	57.8	****	****
mean BP	mmHg	93.2	(12.5)	87.6	94.5	102.0	104.0	82.7	86.7	96.2	101.0	****	****
Wall/lumen	no units	0.2	(0.1)	0.2	0.2	0.2	0.2	0.2	0.2	0.2	0.2	****	****
Vascular mass	mg/mm	11.0	(3.5)	9.2	10.5	12.8	16.4	8.4	9.3	10.8	14.1	****	****
normalized PWV	cm/s/mmHg	7.2	(2.1)	5.9	6.6	7.3	8.9	6.0	6.8	7.5	9.2	****	***
EKG variables													
QTC (QT interval on EKG)	msec	38.9	(2.8)	37.5	37.9	38.7	39.4	38.6	39.0	39.5	40.3	****	****
PR (PR interval on EKG)	msec	15.5	(2.9)	14.8	16.0	16.1	17.3	14.5	15.0	15.4	16.1	****	****
Sonographic variables													
PSV (peak systolic velocity)	cm/s	97.8	(27.9)	137.0	106.0	88.5	75.1	118.0	98.2	82.1	69.1	****	****
EDV (end diastolic velocity)	cm/s	24.6	(6.4)	28.5	25.6	23.4	18.3	27.8	26.7	24.5	18.7	****	**
IP (pulsatility index)	no units	1.9	(0.5)	2.5	2.2	1.9	2.0	2.1	1.8	1.6	1.7	****	****
SD_ratio	no units	4.1	(1.1)	4.9	4.3	3.9	4.3	4.4	3.8	3.5	3.9	****	****
AT (acceleration time)	ms	73.7	(26.0)	73.6	70.7	70.2	79.4	68.6	67.0	74.4	91.6	****	
vti (integral time velocity)	cm	35.7	(9.1)	43.6	37.6	34.1	29.3	38.9	36.6	34.3	28.4	****	****
**Psychological Traits**													
NEO N	NEUROTICISM	90.3	(19.2)	85.9	82.4	85.5	82.6	98.2	93.2	93.7	94.3	****	****
NEO E	EXTRAVERSION	107.0	(16.2)	114.0	111.0	106.0	103.0	113.0	107.0	102.0	99.1	****	****
NEO O	OPENNESS TO EXPERIENCE	104.0	(17.4)	106.0	104.0	99.4	93.0	116.0	109.0	102.0	92.9	****	****
NEO A	AGREEABLENESS	119.0	(15.2)	108.0	114.0	117.0	124.0	115.0	122.0	124.0	129.0	****	****

	Overall		Males				Females				Significant Variation	
**Age Band**			14-29 yrs	30 - 44 yrs	45 - 59 yrs	60-102 yrs	14-29 yrs	30 - 44 yrs	45 - 59 yrs	60-102 yrs	Age	Sex
**# individual¶**	6111	(43.6)	688.3	743.0	611.7	564.2	904.8	1049.6	851.3	698.2		
NEO C CONSCIENTIOUSNESS	121.0	(16.8)	116.0	124.0	123.0	127.0	115.0	122.0	123.0	124.0	****	****
NEO N1 ANXIETY	18.0	(4.9)	16.0	15.9	16.5	15.4	19.6	19.2	19.4	20.1		****
NEO N2 ANGRY HOSTILITY	14.2	(4.3)	13.7	13.3	14.4	14.1	14.5	14.0	14.5	15.0	****	****
NEO N3 DEPRESSION	14.9	(5.2)	13.3	12.9	13.9	14.6	16.0	15.1	16.2	16.8	****	****
NEO N4 SELF-CONSCIOUSNESS	15.4	(4.4)	14.5	14.4	14.8	14.5	16.2	16.0	16.1	15.9		****
NEO N5 IMPULSIVENESS	14.9	(4.1)	16.2	14.9	14.7	12.9	17.2	15.3	14.0	12.7	****	*
NEO N6 VULNERABILITY	12.8	(4.2)	12.2	10.9	11.4	11.1	14.5	13.7	13.4	13.8	****	****
NEO E1 WARMTH	22.1	(3.7)	21.5	22.1	21.8	22.5	22.2	22.5	22.3	22.2	**	****
NEO E2 GREGARIOUSNESS	18.7	(4.6)	19.3	18.7	17.7	17.7	20.6	18.9	17.8	17.6	****	***
NEO E3 ASSERTIVENESS	14.6	(3.9)	15.7	15.9	15.2	14.4	14.9	14.0	13.8	13.5	****	****
NEO E4 ACTIVITY	18.6	(3.7)	18.4	19.3	18.4	17.9	18.0	18.9	18.7	18.6	****	
NEO E5 EXCITEMENT-SEEKING	15.2	(4.9)	19.4	17.2	15.6	13.9	17.4	13.6	12.6	11.5	****	****
NEO E6 POSITIVE EMOTIONS	18.2	(4.7)	19.7	18.4	17.0	17.0	20.2	18.9	17.0	15.8	****	
NEO O1 FANTASY	17.2	(4.9)	18.2	17.2	15.9	14.3	20.3	18.3	16.1	14.2	****	****
NEO O2 AESTHETICS	18.7	(4.6)	17.0	18.0	18.1	17.8	20.4	19.6	19.0	17.8	****	****
NEO O3 FEELINGS	18.8	(3.8)	18.7	18.5	17.7	17.0	20.9	19.4	18.3	18.1	****	****
NEO O4 ACTIONS	16.3	(3.7)	17.1	16.0	15.0	14.6	17.8	17.0	15.9	15.2	****	****
NEO O5 IDEAS	16.3	(5.0)	17.0	17.2	16.3	14.1	17.9	16.9	16.0	13.2	****	
NEO O6 VALUES	17.2	(3.8)	18.2	17.6	16.3	15.3	18.9	18.1	16.4	14.5	****	*
NEO A1 TRUST	18.3	(4.3)	17.0	18.2	18.8	19.2	17.0	18.6	19.1	18.9	****	
NEO A2 STRAIGHTFORWARDNESS	20.2	(4.5)	17.1	18.5	19.6	21.2	19.7	21.3	21.5	22.4	****	****
NEO A3 ALTRUISM	22.6	(3.5)	21.4	21.7	21.8	22.6	22.9	23.3	23.3	23.5	****	****
NEO A4 COMPLIANCE	16.3	(4.5)	14.1	15.6	16.3	17.8	14.2	16.5	17.7	19.0	****	****
NEO A5 MODESTY	19.8	(3.9)	17.9	18.9	19.2	20.5	19.3	20.2	20.4	22.0	****	****
NEO A6 TENDER-MINDEDNESS	21.7	(3.6)	20.1	20.8	21.0	22.3	21.9	22.0	22.0	23.4	****	****
NEO C1 COMPETENCE	19.5	(3.4)	18.9	20.3	20.0	20.9	18.6	19.3	19.3	19.8	****	****
NEO C2 ORDER	18.3	(4.1)	17.6	18.3	18.2	18.3	17.9	18.7	18.5	18.7	****	**
NEO C3 DUTIFULNESS	23.4	(3.8)	21.6	23.6	23.8	24.2	22.0	23.8	24.2	24.3	****	**
NEO C4 ACHIEVEMENT STRIVING	19.5	(3.8)	19.4	20.2	19.5	20.4	19.2	18.9	19.2	19.7	****	****
NEO C5 SELF-DISCIPLINE	21.0	(4.0)	20.2	21.8	21.2	21.7	19.8	21.3	21.1	20.9	****	****
NEO C6 DELIBERATION	19.6	(4.5)	18.3	20.1	20.3	21.5	17.7	19.4	20.1	21.1	****	****

¶: Mean and SD of sample size are calculated based on 60 traits, excluding PSA (2604), TSH (3461), HbA1C (3495), 35 psychological traits (5657)
*: p-value < 0.05
**: p-value < 0.01
***: p-value < 0.001
****: p-value < 0.0005
