# Supplementary material for: Heritability of Cardiovascular and Personality Traits in 6,148 Sardinians
Source: PLoS Genet. 2006 Aug 25;2(8):e132. doi: 10.1371/journal.pgen.0020132 (PMC1557782; doi:10.1371/journal.pgen.0020132)
Supplement: Table S2 — Highlights subsets of traits identified in the clustering analysis, for which the genetic correlation exceeds 0.5. (7 KB PDF) [file pgen.0020132.st002.pdf]

Supplementary Table 2: Clusters when average genetic correlation > 0.5 is used as a cutoff

		Genetic Correlation		
Cluster	Traits	Average	Minimum	Maximum
1	ALT AST	0.528	0.528	0.528
2	CHOLESTEROL LDL	0.907	0.907	0.907
3	LY NE	0.946	0.946	0.946
4	BILIRUBIN_fractionated BILIRUBIN_total	0.749	0.749	0.749
5	IP SD_ratio	0.944	0.944	0.944
6	IMT Wall_Lumen	0.534	0.534	0.534
7	normalized_PWV PWV	0.797	0.797	0.797
8	NEO_A3 NEO_A4	0.564	0.564	0.564
9	NEO_C4 NEO_E4	0.775	0.775	0.775
10	EDV PSV vti	0.539	0.406	0.614
11	NEO_A2 NEO_A5 NEO_A6	0.574	0.493	0.661
12	Hb MCH MCV RBC	0.724	0.350	0.957
13	diam_D diam_S Vascular_mass	0.856	0.792	0.971
14	BMI HIP WAIST WEIGHT	0.849	0.808	0.939
15	NEO_O1 NEO_O2 NEO_O3 NEO_O5	0.751	0.684	0.810
16	AT diastolic_BP mean_BP pulse_pressure systolic_BP	0.617	0.177	0.931
17	NEO_C1 NEO_C2 NEO_C3 NEO_C5 NEO_C6	0.623	0.436	0.778
18	NEO_E1 NEO_E3 NEO_E6 NEO_N1 NEO_N2 NEO_N3 NEO_N4 NEO_N6	0.618	0.233	0.893
