# Supplementary material for: Heritability of Cardiovascular and Personality Traits in 6,148 Sardinians
Source: PLoS Genet. 2006 Aug 25;2(8):e132. doi: 10.1371/journal.pgen.0020132 (PMC1557782; doi:10.1371/journal.pgen.0020132)
Supplement: Table S3 — The table presents Procrustes-rotated principal components from the genetic correlations among the 30 facets of the NEO-PI-R, targeted to the American normative factor structure. (11 KB PDF) [file pgen.0020132.st003.pdf]

Supplementary Table 3 - Genetic Factor Structure of Personality Traits

| NEO-PI-R Facet | N | E | O | A | C | VCC[a] |
|---|---|---|---|---|---|---|
| N1: Anxiety | **.87** | -.02 | -.22 | .09 | .08 | .94 |
| N2: Angry Hostility | **.79** | -.19 | -.25 | -.35 | -.01 | .91 |
| N3: Depression | **.81** | -.16 | -.32 | -.01 | -.32 | .93 |
| N4: Self-Consciousness | **.71** | -.25 | -.35 | .00 | -.34 | .93 |
| N5: Impulsiveness | **.45** | .18 | .36 | -.31 | -.41 | .86 |
| N6: Vulnerability | **.82** | -.07 | -.27 | .20 | -.48 | .97 |
| | | | | | | |
| E1: Warmth | -.20 | **.72** | .21 | .45 | .28 | .99 |
| E2: Gregariousness | -.35 | **.71** | -.21 | .17 | -.03 | .93 |
| E3: Assertiveness | -.33 | **.51** | .44 | -.21 | .42 | .96 |
| E4: Activity | .13 | .47 | .18 | -.23 | **.65** | .96 |
| E5: Excitement Seeking | -.01 | **.67** | .04 | -.43 | -.11 | .99 |
| E6: Positive Emotions | -.40 | **.67** | .39 | .10 | .21 | .88 |
| | | | | | | |
| O1: Fantasy | -.03 | .44 | **.78** | -.02 | -.18 | .89 |
| O2: Aesthetics | .06 | .42 | **.70** | .14 | .02 | .87 |
| O3: Feelings | .10 | .41 | **.81** | .07 | .19 | .90 |
| O4: Actions | -.24 | .31 | **.51** | -.01 | -.23 | .94 |
| O5: Ideas | -.15 | .25 | **.80** | .02 | -.03 | .91 |
| O6: Values | -.28 | -.14 | **.70** | -.01 | -.16 | .93 |
| | | | | | | |
| A1: Trust | -.41 | .36 | .19 | **.55** | .00 | .98 |
| A2: Straightforwardness | .08 | -.17 | .10 | **.82** | .28 | .96 |
| A3: Altruism | .11 | .37 | .03 | **.71** | .39 | .94 |
| A4: Compliance | -.20 | .17 | -.28 | **.69** | -.01 | .88 |
| A5: Modesty | .20 | -.41 | -.19 | **.76** | .04 | .95 |
| A6: Tender-Mindedness | .28 | .23 | .53 | **.73** | -.09 | .88 |
| | | | | | | |
| C1: Competence | -.41 | .36 | .05 | .15 | **.62** | .96 |
| C2: Order | -.08 | .12 | -.35 | -.02 | **.69** | .98 |
| C3: Dutifulness | -.05 | .04 | .02 | .35 | **.80** | .97 |
| C4: Achievement Striving | -.25 | .25 | -.01 | -.36 | **.78** | .94 |
| C5: Self-Discipline | -.35 | -.05 | .01 | .18 | **.86** | .95 |
| C6: Deliberation | -.39 | .07 | -.43 | .22 | **.64** | .79 |
| | | | | | | |
| Congruence coefficient[b] | .94 | .89 | .87 | .96 | .97 | .93 |

*Note.* These are Procrustes-rotated principal components from the genetic correlations among the 30 facets of the NEO-PI-R, targeted to the American normative factor structure. The largest loading for each facet is given in boldface. N = Neuroticism. E = Extraversion. O = Openness. A = Agreeableness. C = Conscientiousness. VCC = Variable congruence coefficient. [a]Total congruence coefficient given in last row. [b]Factor congruence coefficient with target matrix.

This analysis tests the hypothesis that the genetic structure mirrors the phenotypic structure of personality traits; targeted rotation offers a more direct test of this hypothesis than cluster analysis and provides quantitative measures of the phenotypic structure replicability at genetic level. As indicated by the high factor congruence coefficients ( > .85), the genetic structure replicates the phenotypic factor structure well. This analysis suggests that the phenotypic covariation of personality traits is genetically rooted, or in other words, there are shared genes that underlie the facets that define each factor.
